# Steps towards a computational ethology: an automatized, interactive setup to investigate filial imprinting and biological predispositions

**DOI:** 10.1007/s00422-021-00886-6

**Published:** 2021-07-17

**Authors:** Mirko Zanon, Bastien S. Lemaire, Giorgio Vallortigara

**Affiliations:** Center for Mind/Brain Sciences, University of Trento, Rovereto, Italy

**Keywords:** Imprinting, Automated setup, Matlab, Chicks, Predispositions, Innate behaviour

## Abstract

Soon after hatching, the young of precocial species, such as domestic chicks or ducklings, learn to recognize their social partner by simply being exposed to it (*imprinting* process). Even artificial objects or stimuli displayed on monitor screens can effectively trigger filial imprinting, though learning is canalized by spontaneous preferences for animacy signals, such as certain kinds of motion or a face-like appearance. Imprinting is used as a behavioural paradigm for studies on memory formation, early learning and predispositions, as well as number and space cognition, and brain asymmetries. Here, we present an automatized setup to expose and/or test animals for a variety of imprinting experiments. The setup consists of a cage with two high-frequency screens at the opposite ends where stimuli are shown. Provided with a camera covering the whole space of the cage, the behaviour of the animal is recorded continuously. A graphic user interface implemented in Matlab allows a custom configuration of the experimental protocol, that together with Psychtoolbox drives the presentation of images on the screens, with accurate time scheduling and a highly precise framerate. The setup can be implemented into a complete workflow to analyse behaviour in a fully automatized way by combining Matlab (and Psychtoolbox) to control the monitor screens and stimuli, DeepLabCut to track animals’ behaviour, Python (and R) to extract data and perform statistical analyses. The automated setup allows neuro-behavioural scientists to perform standardized protocols during their experiments, with faster data collection and analyses, and reproducible results.

## Introduction

Soon after hatching, the young of nidifugous precocial species such as domestic chicks or ducklings can move, perceive and exhibit impressive cognitive abilities, similar to those of adults (Versace and Vallortigara 2015). This made them ideal animal model systems in ethology and neuroscience to study early learning and brain plasticity (Andrew 1991; Rose 2000).

In the first days of life, the young bird can form a strong attachment (*imprinting*) towards the object it is exposed to (Bateson 1974; Bolhuis 1991; Hess 1959; Lorenz 1937; McCabe 2019; Spalding 1873; Vallortigara and Versace 2018). Even artificial objects or images displayed on monitor screens can trigger imprinting in chicks (Rosa-Salva et al. 2018; Santolin et al. 2020; Versace et al. 2017; Wood and Wood 2015); however, animate objects drive the chicks’ attention at first. Chicks instinctively prefer face-like stimuli (Rosa-Salva et al. 2010) and objects which move like living animals (Rosa-Salva et al. 2016, 2018; Vallortigara et al. 2005); those innate preferences influence the development of filial imprinting memory (Lemaire et al. 2021; Miura et al. 2020). As for simple objects, chicks easily imprint on robots (De Margerie et al. 2011; Gribovskiy et al. 2015; Jolly et al. 2016) which shape their future cognitive abilities and activities. For example, it was shown that chicks imprinted with a moving heating hen–robot develop better spatial navigation skills (De Margerie et al. 2011) and are more synchronized in their daily resting-feeding activities (Jolly et al. 2016) than when exposed to an immobile stimulus.

The use of robots, bio-hybrid organisms, mixed societies and biologically controlled artefacts largely boosted the field of ethology, social behaviour but also of human therapy and assistance (see Romano et al. 2019 for a review). For example, different works investigated the behaviour of fish when swimming with robotic replicas (Landgraf et al. 2016; Polverino et al. 2012), mimicking both healthy and anomalous companions (Romano and Stefanini 2021) or different colour pattern fish (Polverino et al. 2013). Other studies investigated the behaviour of flies when facing bio-robotic conspecifics and predators (Polverino et al. 2012; Romano et al. 2021). In all these cases, the robots must be accepted by the animals to establish a mixed society and modulate the animal’s behaviour. In chicks, acceptance is strongly facilitated by the imprinting phenomenon. In this view, we provide a tool to perform experiments on interaction between chicks and artificial stimuli, exploiting the imprinting process.

Imprinting can be studied on its own as a form of learning, i.e. a recognition memory (Bolhuis and Honey 1998; Horn 1985, 1998; Mccabe 2013; Nakamori et al. 2013), but it can also be used as a key to mind, i.e. to investigate aspects of object cognition such as object permanence (Regolin et al. 1995; Vallortigara et al. 1998), number (Lemaire et al. 2020; Rugani et al. 2009, 2010, 2011, 2013, 2017), space (Vallortigara 2015; Vallortigara et al. 2010) and others (for a review see Chiandetti and Vallortigara 2018; Marino 2017; Vallortigara 2012; Vallortigara 2021). In most experimental designs, the imprinting preference can be investigated using a dual free-choice task. The chicks or other young birds (Martinho and Kacelnik 2017) are exposed to the imprinting object on one side and some novel object on the other one (Lemaire et al. 2021; Rugani et al. 2010). By monitoring the animal's first choice and time spent close to the displayed stimuli, researchers can address their experimental questions, whether it is about learning or other cognitive processes using filial imprinting. The dual free-choice task allows for the investigation of biological predispositions too (Rosa-Salva et al. 2016; Versace et al. 2016, 2018).

Traditionally, monitoring the animals’ preferences required scientists to observe the subjects’ behaviour manually or using some automatic device (Izawa et al. 2001; Yamaguchi et al. 2012). It can also be done offline by watching video recordings, a time-consuming activity, prone to biases, primarily when animals are studied for prolonged periods (for example, many hours or days of imprinting). Recent progress in technology can provide more efficient and reliable procedures, allowing for precise behavioural measurements through time while fully controlling the environment and stimuli characteristics. These computational advances and their automation lead to an analysis that is rapid, unbiased, more reliable and reproducible (Anderson and Perona 2014). It is interesting to notice that automation in computational ethology is not limited to data collection and analyses but can be crucial at different levels of an experiment. While some levels have already been automatized (Wood 2013), others are still performed by a human operator. In particular, in imprinting studies, a wholly automated computational protocol is lacking. The main points that have to be covered for running free-choice testing experiments using an imprinting procedure are: (1) the creation of a setup in which animals can freely move and live for extended periods, being presented with artificial stimuli; (2) the design of controlled stimuli, and the schedule of their presentation; (3) the recording of the animal behaviour, with data extraction and analyses; (4) the possibility of direct interaction between the behaviour expressed by animals (e.g. approach, eye-use to look at the stimuli) and coordinated changes in stimulus presentation.

This paper describes a complete automated setup that uses different workflows to measure animal behaviours in a fully automatic manner. We mainly focus on the apparatus (testing cages; much improving a previous model by Wood 2013) and the release of a new program (*ImprintSchedule*) to precisely control stimuli presentation; moreover, we provide information for a completely automated workflow including stimuli creation, animal tracking and data analysis.

## The automated setup

We first describe the apparatus and then provide more details about the automatization procedures for stimuli presentation.

### The apparatus

**Fig. 1:**
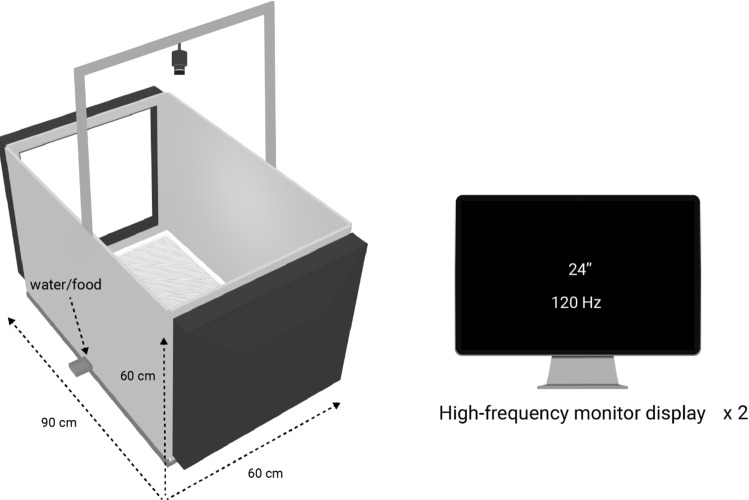
The automated cage. A sketch of the apparatus is shown, with the position of the monitor screens and camera (left). Multiple setups can be used at the same time. Each setup contains a video camera (e.g. Microsoft LifeCam) and two high-frequency monitors (at least 120 Hz)

The apparatus consists of a simple rectangular cage where the chicks can freely move and approach stimuli displayed on screens located at the opposite short ends of the cage (Fig. 1).

The apparatus is 90 cm long, 60 cm wide and 60 cm high. It allows suitable conditions for the animals up to several days according to the standard in UE. Water and food are located on the sides, equally distant from both screens; they are available ad libitum and can be refilled from the outside without interfering with the animal. The chick’s behaviour is continuously recorded using a camera located 105 cm above the ground; good quality video recordings (e.g. a Microsoft LifeCam with a minimum resolution of 640–480 pixels and sufficient lightning conditions) are essential to facilitate data extraction.

The main elements of the apparatus are the two opposite screens. They are used to present the stimuli to the animal and are the only source of illumination in the environment. This is an important factor to control because lighting can create flickering effects disturbing the animals (Inger et al. 2014). For instance, domestic chicks perceive light as a constant stream when the frequency reaches 115 Hz (Lisney et al. 2011, 2012). Therefore, in our setup, we use high-frequency monitors (ASUS MG248QR, 120 Hz). As for the light, the frame frequency at which the stimuli are displayed must be controlled to ensure a smooth perception. We display them at 120 frames per second as well.

Combined with the use of visually naïve animals (Versace and Vallortigara 2015)—chicks are kept in the dark until the experiment starts—the exploitation of this setup can exclude the effect of specific experience on the animal preferences for both short and long periods. To automate the process of stimuli presentation we created *ImprintSchedule*, a user-friendly interface that allows scientists to plan their experiments, from a few minutes/hours to several days of exposure/testing.

### Stimuli presentation

**Fig. 2:**
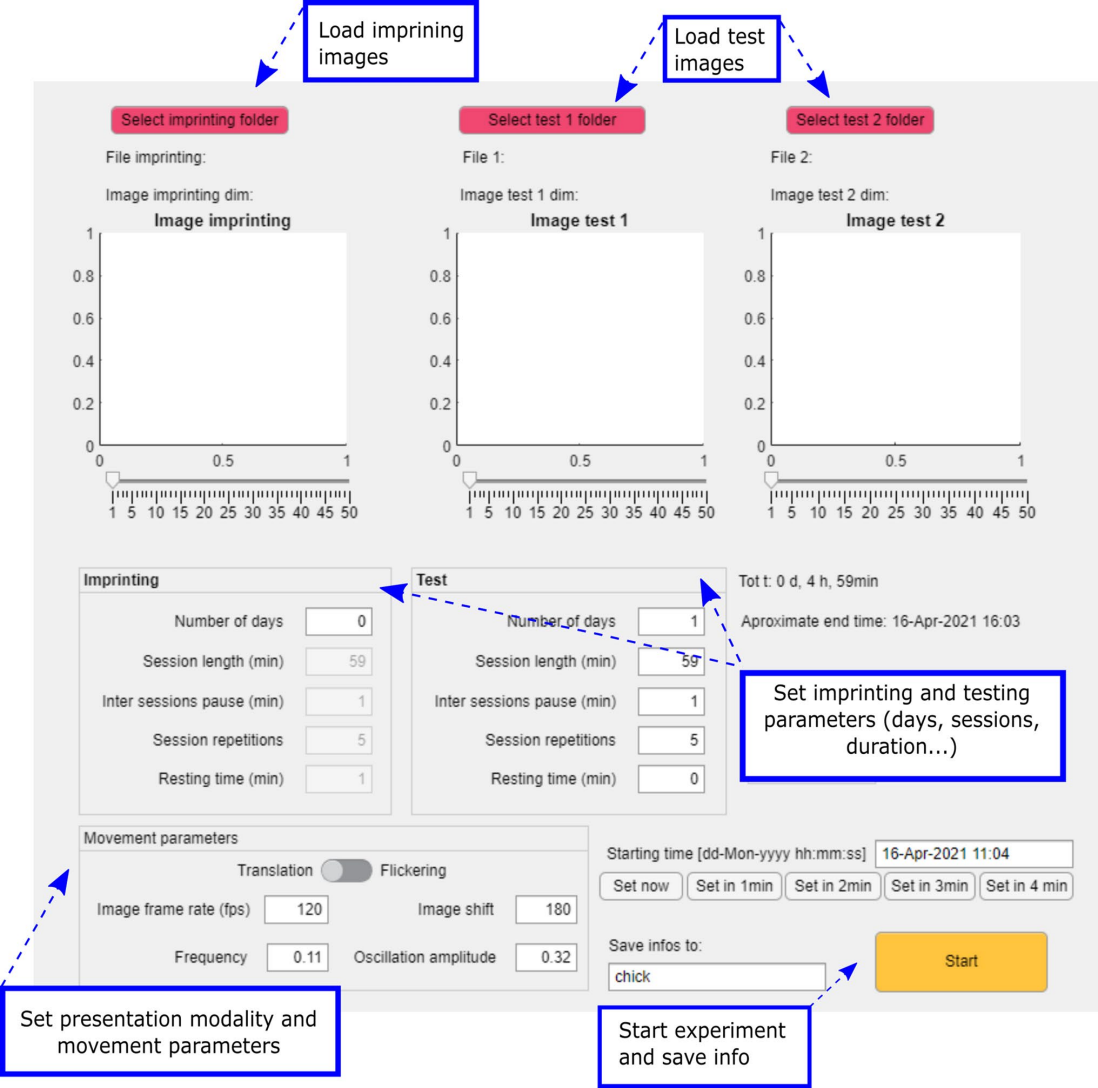
*ImprintSchedule* graphic user interface. Different parameters of image presentation can be set, such as the duration of image appearance (divided into days and sessions) both in the imprinting phase and in the test phase, and the presentation modality (e.g. translatory or flickering) with all the relative motion settings

The second important element contributing to this setup is an automatized presentation method, to display stimuli on-screen with a defined schedule. This might appear trivial when animals are tested for short durations but can get laborious in long-lasting experiments (Lemaire et al. 2020; Wood 2017). In a recent study (Lemaire et al. 2021), we imprinted several chicks with different objects and tested their filial preferences for 6 days. The stimuli were displayed thanks to videos rendered in Blender and a video media player executing handmade playlists lasting 6 days. This task was extremely time-consuming, erring for the experimenter, hard to duplicate in small-space laboratories, as well as computationally heavy. Contrariwise, having a tailored-made program handling the stimuli presentation automatically would make this experimental process fast, reliable, and easy to replicate in other laboratories. Therefore, we developed *ImprintSchedule*, a graphic user interface written in Matlab (Matlab R2019a, The MathWorks Inc., Natick, Massachusetts, USA) and exploiting Psychtoolbox-3 (the Psychophysics Toolbox extensions; Brainard 1997; Pelli 1997) to set and control screen presentation (Fig. 2).

This tool allows researchers to create their custom protocol of stimuli presentation to imprint and test animals, in a versatile but standardized and precise way without requiring computational skills. Our tool can help duplicate experimental designs studying or using imprinting (such as the one briefly described above).

The development of filial preferences has been primarily investigated in the few minutes of stimuli exposure (Bolhuis 1991). We have recently studied the development of those preferences after days of exposure (Lemaire et al. 2021). Our application could help researchers further explore the development of filial preferences with better control of the stimuli appearances and disappearances. More than that, all sorts of experimental paradigms that require controlling stimuli presentation on screens could be carried out using our application. This is described below.

Briefly, three different groups of PNG images can be loaded in our *ImprintSchedule*: the first group represents what we call the ‘imprinting set’, while the other two are the ‘test sets’. The images loaded within the ‘imprinting set’ are presented to the animal one at a time on one of the two screens, while the other is dark. The images loaded within the two test sets are displayed simultaneously, one per screen, allowing the animal to freely approach either one of them. Depending on the side the animal moves to, its choice can be monitored and measured. In imprinting paradigms, this phase aims to measure whether imprinting occurred by giving the animal a choice between its imprinting stimulus and a new one (see Lemaire et al. 2021; Miura and Matsushima 2016; Versace et al. 2017 for different examples of dual choice tasks using imprinting that could be performed using our program). Note that in the absence of an imprinting phase, the images loaded in the test sets can also be used to investigate the animals’ spontaneous preferences (see Rosa-Salva et al. 2015, 2021 for reviews).

All the settings are adjustable, and the experimenter can choose the duration of images displayed during the single (imprinting) and dual (test) presentation phases. In long-lasting experiments, constant exposure to the stimuli could be detrimental, thus adding some break, i.e. by turning the screens dark, would allow the animals to rest, helping memory formation and consolidation (Jackson et al. 2008). For this reason, the presentation can be split into different steps, reported as days and sessions: the number of days and sessions per day can be set up, like the inter-trial intervals between them. If multiple images are loaded in one set (to present different stimuli during each phase), a new random stimulus image will be displayed during each different session. The order of presentation of the stimuli and the timing and screen positions are saved in an Excel file at the end of the experiment. This allows precise monitoring of all experimental parameters, checking how these influence the animal’s performance. To minimize any position bias, all the presentations on the two different screens can be controlled in a pseudo-random way, balancing the amount of time the same set of stimuli is presented on each side.

Since motion attracts the chick’s attention (Bolhuis 1991), our program can display moving stimuli, creating a perceptively richer artificial environment. We implemented two different movements: a translatory motion, which consists of a horizontal oscillation of the image with a speed following a sinusoidal function (from which the amplitude and period of the oscillation can be controlled); a flickering motion, which consists of an appearance/disappearance of the image with user-defined timing. Even the vertical position of the image can be adjusted.

*ImprintSchedule* gives more flexibility in building experimental designs requiring single or dual images presentations. It is originally built for imprinting and spontaneous choice tests paradigms but can be used for other applications too.

## Complete workflow

The setup can be implemented into a complete automatized workflow, starting from the imprinting and test phases, ending up with an accurate analysis of the chick’s behaviour. To perform such a complete experiment, some other steps are missing from the previous discussion: in the following we will give more information, to explain our usual procedures during free-choice experiments with chicks; still, other approaches are possible, implementing the use of our testing cage with *ImprintSchedule*.

### Stimuli creation

**Fig. 3:**
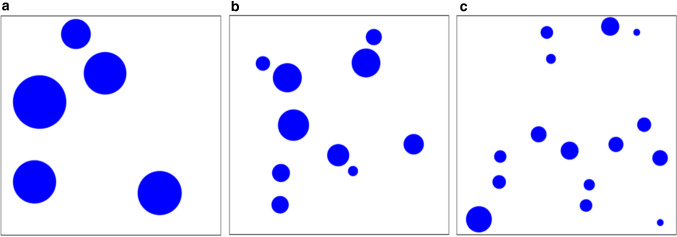
Example of artificial numerical stimuli with controlled characteristics. While in nature the total area of similar elements is covarying with the numerosity, we can create controlled stimuli (for example, with *GeNEsIS*, Zanon et al. 2021) in which the total area (TA) is perfectly varying opposite to the number of elements (*n*). In this case, we have three examples with **a** *n* = 5, TA = 600 px^2^; **b** *n* = 10, TA = 300 px^2^; **c** *n* = 15, TA = 200 px^2^. The precise control over specific physical variables (even in an unnatural way) allows us to interpret their role in the chick’s perception

The first desirable characteristic to control for is the stimulus itself: this is the main and most influential element the animal is going to experience. Depending on the kind of experimental question, simple photographs of conspecifics or sketches of natural elements could be potential stimuli to test the birds with. Nevertheless, these kinds of stimulations lack specific controls over different physical parameters, affecting the accuracy we could obtain with a computational approach. Exploiting the automated setup and the presentation on screen, specific images can be created by tuning their physical characteristics in a parametrized way. An example is provided by studies on numerosity cognition. It is well known how numerosity discrimination could be affected by continuous variables (like total area, the density of elements, the contour length of the stimulus and so on) that co-vary with the numerosity itself (see Leibovich et al. 2017; Lorenzi et al. 2021 for general reviews). With a computational approach, we can create stimuli for which these variables are controlled. Recently, we have developed a software to create in a standardized way these kinds of stimuli, that can be integrated within this workflow (Zanon et al. 2021). This approach allows us to deeply study the relation between animals’ behaviour and artificial stimulation, even beyond the usual limits imposed by natural stimuli. For example, one could create numerical arrays (i.e. pictures of dots with different numerosity, areas and spatial distribution) manipulating physical variables in unconventional ways (e.g. letting the total size of elements decrease while the numerosity is increasing, and so on; Fig. 3).

Another simple example on this topic could involve experiments to investigate whether chicks would care more about change in numerosity or change in others continuous physical variables. In this case, our apparatus could be used to perform a spontaneous dual choice task, presenting the chick with artificial numerical stimuli. Just after hatching the young bird can be put inside the automated cage facing the two moving stimuli at the opposite side of the apparatus. At this point, different images can be presented, either a sequence of stimuli with the same numerosity but different continuous physical variables or a sequence of stimuli changing in the number of elements but with constant physical variables (Zanon et al. 2021). Since the chicks can be kept in the dark until the testing session and the stimuli are the first objects they see, the animals could recognize them as socially relevant and approach them on the monitor screens. Monitoring the time the animals spent near to each screen, we can analyse whether chicks present a higher preference for a change in numerosity or a change in the continuous physical variables, measuring which one of these different features could be more relevant to them. The creation of such virtual environments may help researchers to develop strict and precise control over different variables, monitoring all image parameters and timing of stimulus presentation. This would not be doable with naturalistic stimuli or manual approaches, showing all the benefits of such an implementation exploiting the animal–artificial stimulus interaction exploited by our setup.

### Animal tracking and data analysis

**Fig. 4:**
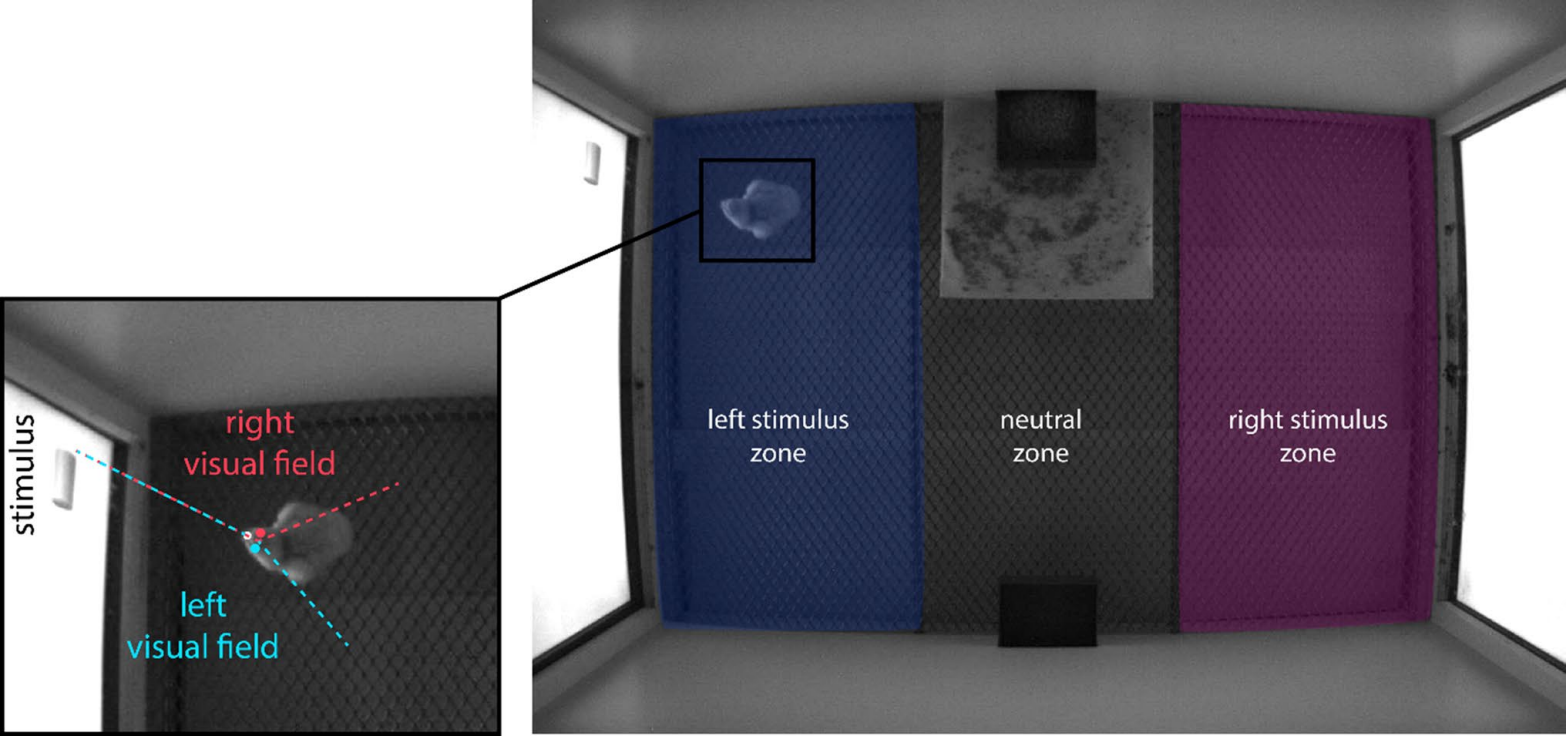
Using *Visual field Analysis* (VFA, Josserand et al. n.d.; Josserand and Lemaire 2020), the location of the chicks is monitored and the number of seconds in different zones is measured. VFA also analyses the eye used to observe the stimuli displayed on the screens and the motoric activity of the animal’s head. Here, the chick is located in the left stimulus zone and observes the stimulus binocularly (mainly using its left visual field)

Another fundamental step in our computational approach concerns the chicks’ behaviour analysis. Even here, a manual estimation of the time spent by the animal close to a specific stimulus or a mechanical evaluation of the subject choice is time-consuming and could be a source of error and bias. After recording the chicks’ movement inside the cage, the videos can be instead automatically analysed by an artificial neural network, extracting the position of different body parts for each frame. We routinely performed this with DeepLabCut (Mathis et al. 2018; Nath et al. 2019), a powerful tool largely used in computational ethology studies (Labuguen et al. 2021; Worley et al. 2019; Wu et al. 2020). After positions’ extraction, a CSV file with all the body parts coordinates is available, from which a whole statistical analysis is coded. For example, it is possible to calculate the number of frames the chick spends in a specific area of the cage, convert them in time, and compare the time spent close to the two stimuli to obtain a preference score. We developed a program (VFA, *Visual Field Analysis*) to automatically assess the time spent in different areas, the motoric activity and the eye used by the animal to look at the stimuli, making the data analysis quick and reliable (Fig. 4; Josserand et al. 2021; Josserand and Lemaire 2020).

### Future perspectives

We have implemented and described a powerful workflow, starting from controlled image presentation and ending up with automated tracking and data analysis for studies on imprinting and associated phenomena, a classical topic in ethology. We want to stress the possibility of using this setup in a closed-loop configuration, implementing online animal tracking triggering image presentation. With such a tool we could move deeper in the investigation of animal interaction with the artificial stimulus. Implementing a computational protocol to continuously extract the animal position while the experiment is running, and analysing it online, it is possible to directly synchronize the presentation script with the animal’s positions, triggering image display with the animal movements. Stimuli can be created directly interacting with the chick: not only the subject is interacting with the stimuli on screen, but the stimuli themselves can react to the animal’s behaviour, allowing interesting possibilities for research on social behaviour. This approach, working on two-way animal–robot interaction, can complement others already existing for different, smaller species, e.g. fish (Kim et al. 2018).

For example, we have previously described how with our program GeNEsIS (Zanon et al. 2021) we can generate artificial images with unconventional statistical regularities, to study chicks’ predisposition to them. With this closed-loop approach, we can push the idea further: we can, for example, create stimuli maintaining a fixed visual angle, adapting their dimensions proportionally to the distance of the chick from the screen. This would allow rearing naïve animals in virtual worlds to test the role of experiential and innate factors in the development of behaviour.

Another example comes from studies on biological motion perception (Johansson 1973). It has been shown that newly hatched chicks facing a set of moving points arranged as the main junctions of a hen—and thus mimicking hen’s motion (Miura and Matsushima 2012, 2016; Vallortigara et al. 2005; Vallortigara and Regolin 2006)—tend to align themselves to the direction dictated by this virtual stimulus: if the moving points hen is changing direction, the chick is doing it as well. Such an experiment could now be conducted with our automated cage within a closed loop in which the artificial hen would change direction contingent on the chick’s movements. This would improve current approaches in which a one-way interaction (animal–stimulus) is present, studying a two-way interaction which would improve the behavioural readout, triggering a richer palette of scenarios in chicken approach responses.

This sort of implementation would push further the study of animal behaviour in laboratory conditions, analysing in depth all the relevant characteristics of the interaction animal–artificial stimulus.

## Conclusions

We presented an automated setup to perform imprinting and dual choice tasks on chicks in laboratory conditions. With its relatively big dimensions and high-frequency screens, the apparatus allows the chicks to freely move in a homogeneous and neutral environment, approaching well-displayed conspicuous stimuli (Fig. 1). This setup allows both short- and long-time experiments, recording a big amount of behavioural data on naïve chicks.

The provided software, *ImprintSchedule*, has a graphic user interface that helps the researchers, even without coding capabilities, to schedule a standardized and precise experiment presenting images on two opposite screens. This tool could serve as a base instrument, in the direction of a common method for imprinting and dual choice tasks experiments on chicks; studies performed in this way can be easily replicated, setting the proper parameters (image motion modality, the timing of image presentation and pause, number of sessions etc.; see Fig. 2).

Moreover, with a computational approach to image creation (for example, using software that generates parameterized images like our *GeNEsIS*, Zanon et al. 2021) we can have strict control over specific physical characteristics presented to the naïve chicks, monitoring how the animal interacts with these artificial stimuli. This approach could open up the way to a detailed study of a huge variety of physical characteristics and how relevant they are considered by chicks interacting with them.

Adding an automated tracking of the animal behaviour (e.g. using artificial neural networks, like in DeepLabCut) and computational statistical analysis, we proposed here a complete workflow that not only facilitates the researchers work but also improves the precision of the research, reducing experimenter biases and facilitating the reproducibility of the experiments.

One additional advantage of using the automated apparatus is the increased productivity: with multiple setups in parallel, many chicks can be tested at the same time. Still, this configuration might not be suitable for all the experimental designs, especially the ones that are cognitively demanding. Although chicks cannot see each other, they probably can still hear one another if all the different cages are located in the same room. It is still unclear how this could influence the animal’s aptitude to approach a stimulus or not; further studies should be conducted in this direction.

Future implementations could be directed towards a closed-loop approach, in which the stimuli presentation is directly triggered by the animal behaviour. With this configuration it would be possible to push further our investigation of the interaction animal–stimulus, creating artificial controlled elements changing their characteristics in response to animal behaviour.

In conclusion, we hope this instrument could be used to further study, in a standardized way, how chicks are predisposed, interact and elaborate specific physical characteristics that can be generated in a controlled way through artificial stimuli; moreover, to perform research that is more powerful, leading to stronger statistics and replicable results.

## Data Availability

All the reported materials are freely available at https://github.com/MirkoZanon/ImprintSchedule, https://github.com/MirkoZanon/GeNEsIS and https://github.com/mathjoss/VisualFieldsAnalysis.
